# PP2A Regulates Phosphorylation-Dependent Isomerization of Cytoplasmic and Mitochondrial-Associated ATR by Pin1 in DNA Damage Responses

**DOI:** 10.3389/fcell.2020.00813

**Published:** 2020-08-28

**Authors:** Yetunde Makinwa, Brian M. Cartwright, Phillip R. Musich, Zhengke Li, Himadri Biswas, Yue Zou

**Affiliations:** ^1^Department of Cancer Biology, The University of Toledo College of Medicine and Life Sciences, Toledo, OH, United States; ^2^Department of Biomedical Sciences, JH Quillen College of Medicine, East Tennessee State University, Johnson City, TN, United States

**Keywords:** ATR, Pin1, PP2A, UV irradiation, DNA damage response, ATR antiapoptotic activity at mitochondria, BID

## Abstract

Ataxia telangiectasia and Rad3-related protein (ATR) is a serine/threonine-protein kinase of the PI3K family and is well known for its key role in regulating DNA damage responses in the nucleus. In addition to its nuclear functions, ATR also was found to be a substrate of the prolyl isomerase Pin1 in the cytoplasm where Pin1 isomerizes *cis* ATR at the Ser428-Pro429 motif, leading to formation of *trans* ATR. *Cis* ATR is an antiapoptotic protein at mitochondria upon UV damage. Here we report that Pin1’s activity on *cis* ATR requires the phosphorylation of the S428 residue of ATR and describe the molecular mechanism by which Pin1-mediated ATR isomerization in the cytoplasm is regulated. We identified protein phosphatase 2A (PP2A) as the phosphatase that dephosphorylates Ser428 following DNA damage. The dephosphorylation led to an increased level of the antiapoptotic *cis* ATR (ATR-H) in the cytoplasm and, thus, its accumulation at mitochondria *via* binding with tBid. Inhibition or depletion of PP2A promoted the isomerization by Pin1, resulting in a reduction of *cis* ATR with an increased level of *trans* ATR. We conclude that PP2A plays an important role in regulating ATR’s anti-apoptotic activity at mitochondria in response to DNA damage. Our results also imply a potential strategy in enhancing cancer therapies *via* selective moderation of *cis* ATR levels.

## Introduction

ATR is a phosphatidylinositol 3 kinase (PI3K)-like protein that plays a crucial role in sensing DNA damage for maintenance of genomic integrity (Zhou and Elledge, 2000; Zou and Elledge, 2003). It is an essential factor for cellular regulation of DNA damage responses in order to maintain cell homeostasis. Following DNA damage ATR, together with its nuclear partner ATR-interacting protein (ATRIP), senses and recognizes the presence of elongated regions of replication protein A (RPA)-coated ssDNA resulting from DNA damage-induced replication stress. This recognition allows activation of the DNA damage checkpoint responses, including cell cycle arrest, gene expression alterations, DNA repair and/or apoptosis (Zou and Elledge, 2003; Sancar et al., 2004; Mordes and Cortez, 2008; Flynn and Zou, 2011; Nam and Cortez, 2011; Awasthi et al., 2016; Saldivar et al., 2017; Lecona and Fernandez-Capetillo, 2018). The cell cycle arrest that follows sensing of DNA damage ultimately leads to DNA repair or, if too severe, to apoptotic cell death. ATR also is involved in sensing mechanical stress to the cell (Baumann, 2014; Kumar et al., 2014), replication origin firing (Shechter et al., 2004; Moiseeva et al., 2019) and autophagy (Liu et al., 2018; Ma et al., 2018).

Hilton et al. (2016) reported that ATR has two peptidylprolyl isomeric forms, *cis* and *trans*, in cells. The cell nucleus contains only *trans* ATR, while the relative levels of *cis* and *trans* forms in the cytoplasm are DNA damage dependent. Upon DNA damage, the *trans* isomeric form of ATR in the nucleus is responsible for its DNA damage checkpoint functions. In contrast, the *cis* isomeric form generated in the cytoplasm after DNA damage directly acts at the mitochondria as an antiapoptotic protein. Thus, ATR can directly regulate apoptosis at the mitochondrion, a response distinct from the ATR-mediated DNA damage checkpoint pathway. However, it is believed that the *trans* and *cis* ATR activities in the nucleus and mitochondria, respectively, are coordinated and the balance of the activities plays a critical role in ATR-dependent DNA damage responses. It was reported that the interconversions between *cis*- and *trans*-isomeric forms of ATR are regulated by the prolyl isomerase Pin 1 (peptidylprolyl *cis*/*trans* isomerase NIMA-interacting 1) (Hilton et al., 2016). In cells, Pin1 isomerizes ATR at the motif of Ser428-Pro429, converting the protein from *cis* to *trans*. Upon DNA damage, Pin1 is phosphorylated at S71 which inactivates Pin1, leading to cytoplasmic accumulation of the antiapoptotic *cis* ATR (Hilton et al., 2016). It is believed that upon DNA damage, *trans*-ATR is activated in the nucleus to initiate DNA damage signaling and checkpoint pathways, which lead to cell cycle arrest and promote DNA repair. At the same time, coordinately, *cis*-ATR forms in the cytoplasm and is translocated to mitochondria to prevent activation of Bax, suppressing apoptosis (Hilton et al., 2016). The coordination between *trans*- and *cis*-ATR activities prevents premature cell death while DNA damage is removed, followed by resuming cell cycling.

Pin1 catalyzes the isomerization of specific phosphorylated Ser/Thr-Pro amide bonds in proteins. This phosphorylation-dependent isomerization can lead to major conformational changes in protein structure and function (Lu et al., 1996; Lu, 2000; Liou et al., 2011; Schmidpeter and Schmid, 2015). In the case of ATR, Pin1 recognizes the phosphorylated Ser428-Pro429 (pSer428-Pro429) motif in ATR and isomerizes ATR from the *cis*-isomeric form (ATR-H) to the *trans* form (ATR-L) (Hilton et al., 2016). The fact that Pin1 isomerization of ATR depends on the status of Ser428 phosphorylation suggests a mechanism by which the balance between kinase(s) and phosphatase(s) activities can regulate Pin1 activity toward ATR. While the regulation is expected to have an important effect on the ATR’s antiapoptotic activity at mitochondria and DNA damage responses, the identities of the kinase(s) or phosphatase(s) remain unknown.

Protein phosphatase 2A (PP2A) plays an important role in regulating DNA damage responses (DDR) by dephosphorylating DDR proteins to change the phosphorylation status of proteins that are critical to genome stability (Freeman and Monteiro, 2010; Harris and Bunz, 2010; Palii et al., 2013; Ferrari et al., 2017; Merigliano et al., 2017; Ramos et al., 2019). Substrates of PP2A in DDR include ATR, ATM, DNA-PK, Chk1, Chk2, p53, and so on (Ramos et al., 2019). All these regulations are carried out in the nucleus where DNA damage checkpoint signaling occurs. For example, PP2A activation attenuates ATR/ATR-dependent DDR (Ferrari et al., 2017). Inhibition or knockdown of PP2A leads to an increase of y-H2AX, an important DNA damage signal of DNA strand breaks (Nazarov et al., 2003; Chowdhury et al., 2005; Keogh et al., 2006). PP2A and Wip1 dephosphorylate ATM at S1981 and, thus, suppress ATM activity (Goodarzi et al., 2004; Shreeram et al., 2006). PP2A also is involved in regulating cell death (Andrabi et al., 2007; Guenebeaud et al., 2010; Lee et al., 2011; Sun and Wang, 2012; Zhou et al., 2017).

In this study, we examined how Pin1-mediated ATR isomerization is regulated. Specifically, we identified PP2A, which belongs to the Ser/Thr phosphoprotein phosphatase family, as the phosphatase responsible for the dephosphorylation of ATR at Ser428. We show that when PP2A is inhibited, phosphorylation of ATR at Ser428 significantly increases, promoting the formation of *trans* ATR with less *cis* ATR in the cytoplasm. This reduces the protection of cells from apoptotic death following UV-induced DNA damage. Our study reveals a potential pharmacological target in the regulation of mitochondria-associated ATR by phosphatase PP2A that may imply new therapeutic strategies against diseases involving apoptotic cell death and cancer.

## Materials and Methods

### Cell Culture, UV Irradiation, siRNA, and Inhibitor Treatments

A549 and HCT 116 cell lines were used for all experiments. UV treatments were delivered at 40 J/m^2^, followed by a 2 h recovery, except for the graduated UV doses and timed recoveries as indicated in the text and specific figure legends. The siRNAs targeting PP2A, PP4, and PP5 were directed against the catalytic subunits of PP2Ac, PP4c, and PP5c, respectively. The PP2A inhibitor (PP2Ai) used was LB-100 at a dose of 10 uM concentration for 1 h before and during all UV treatments. The CRISPR-generated knock-in cell lines (ATR-S428A and ATR-P429A) were generated with the human A375 melanoma cell line. Based on off-target profile analysis and distance to target site, the gRNA, GTGATGGAATATCACCCAAANGG was used to create single base substitutions in the ATR gene in A375 cell line to create the mutant A375 cell lines.

### Cell Lysis and Immunoblotting

Cells were harvested by scraping or trypsinization and lysed with buffer [50 mM Tris–HCl, pH 7.8, 150 mM NaCl, 1 mM EDTA, 1% Triton X-100, 1X protease/phosphatase inhibitor cocktail (Thermo 1861280)]. 2X SDS loading buffer was added to the lysates before heating at 95°C for 5 min.

Three to eight percentage Tris-Acetate SDS PAGE gradient gels (Invitrogen EA0378) were used to resolve ATR-H from ATR-L; otherwise, standard SDS PAGE gels were used. PVDF membranes (Amersham 10061-492) were used to capture proteins transferred from gels. Chemiluminescent signal was captured using the GE Amersham Imager 680. Antibodies and their dilutions for western blots include pATR S428 (Cell Signaling 2853) 1:1000, PP2Ac (Cell Signaling 2038) 1:1000, GAPDH (Cell Signaling 5174) 1:1000, PARP 1 (Cell Signaling 9532) 1:1000, Bid (Cell Signaling 2002) 1:1000, or (Santa Cruz Biotechnology sc6538) 1: 500, Beta Actin (Invitrogen MA1-140) 1:5000, mtHSP70 (Invitrogen MA3-028) 1:1000, ATR (Bethyl Laboratories, Inc., A300-137A, A300-138A) 1:8000, ATRIP (ABclonal A7139) 1:1000, PP2A-Cα/β (Santa Cruz Biotechnology sc-80665) 1:500, Cleaved Caspase-3 (Cell Signaling 9664) 1:1000, PP4c (Bethyl Laboratories, Inc., A300-835A) 1:8000, PP5c (Bethyl Laboratories, Inc., A300-909A) 1:8000.

### RNAi and Plasmid Transfections

Transfections were performed using the Polyplus siRNA (409-10) and DNA transfection reagents (101-10) according to manufacturer’s instructions. DNA transfection was into the HCT 116 ATR^flox/–^ cell line.

### Cellular Fractionation

Lysis buffers and differential centrifugation fractionated the cells into cytoplasmic and nuclear isolates at 4°C. 10 volumes of the hypo-osmotic cytoplasmic lysis buffer (10 mM HEPES, pH 7.9, 10 mM KCl, 3 mM CaCl_2_, 1.5 mM MgCl_2_, 0.34 M sucrose, 10% glycerol, 0.1% Triton X-100) with 1X protease and phosphatase inhibitor cocktail was added to resuspend 1 volume of packed cells for 10 min. The suspension was then centrifuged for 7 min at 600 × *g* and the supernatant collected as the cytoplasmic fraction. The pellet (nuclei) was washed twice in ice-cold cytoplasmic lysis buffer, then lysed with 1/10 volume of the nuclear lysis buffer (50 mM Tris–HCl, pH 7.9, 140 mM NaCl, 3 mM CaCl_2_). After rotation for 15 min at 4°C the nuclear lysate was collected as the supernatant after centrifugation at 10,000 rpm for 10 min at 4°C.

To establish equal protein loading and the quality of all fractionation procedures the separation of cytoplasmic GAPDH from nuclear PARP was assessed by western blotting (WB).

### Mitochondrial Isolation

Mitochondrial isolation was performed either by using the Qiagen mitochondrial extraction kit (Qiagen 37612) or according to the established protocol by Frezza et al. (2007). Mitochondrial HSP70 (mtHSP70) protein was used to assess the quality of protein loading from mitochondrial isolates in WB.

### Co-immunoprecipitation Assays

The cytoplasm of A549 cells was isolated after PP2Ai pretreatment (where indicated) and UV irradiation. ATR antibody (Bethyl labs) was added at 1 μg/mL for overnight incubation at 4°C. ATR protein was immunoprecipitated (IPed) with magnetic beads (Pierce) for 3 h at 4°C, and then washed three times in co-IP wash buffer (50 mM Tris–HCl, pH 7.6, 140 mM NaCl, 1 mM EDTA, 10% glycerol, 0.2% Tween-20). The final pellet was resuspended in 1X SDS-loading buffer. The mixture was boiled at 95°C for 5 min. The amounts of Bid and PP2A bound to the pulled-down ATR was determined by WB with Bid and PP2Ac antibodies, respectively (Cell signaling).

### Duolink *in situ* Proximity Ligation Assays

The Duolink protein-protein interaction assay was performed according to the manufacturer’s instructions (Sigma DUO 92101). Images were captured with a Life Technologies EVOS microscope.

### Cellular Viability Assays

MTT assays were performed following the manufacturer’s instructions (Cayman’s MTT Proliferation Assay kit #10009365).

### Statistical Analysis

The statistical analysis of samples was carried out with the student’s *t*-test (two-tailed) and one-way ANOVA. A *p*-value of less than 0.05 was taken as significant.

## Results

### PP2A Dephosphorylates Cytoplasmic ATR-L at Serine 428

**FIGURE 1:**
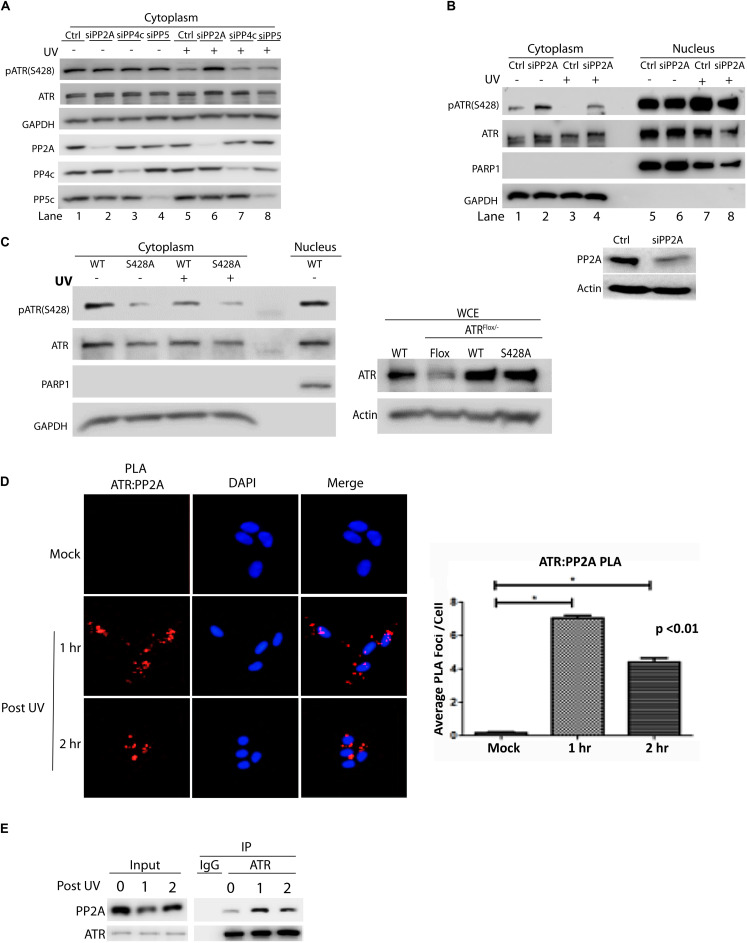
Protein phosphatase 2A (PP2A) specifically dephosphorylates cytoplasmic ATR at S428 and its action involves direct binding to ATR. **(A)** A549 cells were treated with siRNAs against three protein phosphatases (PP2Ac, PP4c, and PP5c) followed by UV treatment at 40 J/m^2^ with a 2 h recovery. Analysis of cytoplasmic extracts reveals that PP2A is required to dephosphorylate cytoplasmic pATR (S428). **(B)** siRNA knockdown of PP2Ac in A549 cells was followed by UV treatment at 40 J/m^2^ with a 2 h recovery. pATR (S428) levels are increased in cytoplasm with PP2Ac knockdown while the phosphorylation status of ATR in the nucleus remains unchanged. WB of whole cell extracts shows PP2A siRNA knockdown efficiency in A549 cells **(C)** HCT 116 ATR^flox/–^ cells were transfected with N-terminal Flag-tagged wild-type (WT) or S428A mutant ATR expression constructs and UV treated at 40 J/m^2^ with a 2 h recovery. Analysis of the cytoplasmic extracts reveals that PP2A specifically targets the pATR (S428) residue. The pATR (S428) observed in the S428A cells reflects some phosphorylation of the residual endogenous ATR remaining in the ATR^flox/–^ cells (right panel). **(D)** PLA revealing that in A549 cells there is a direct interaction between ATR and PP2A, especially after UV irradiation at 40 J/m^2^. A DAPI-staining overlay is used to show location of the nuclei. The bar graph represents a statistical analysis of the PLA images. **(E)** A549 cells treated with UV at 40 J/m^2^ were lysed, followed by co-immunoprecipitation of PP2A using anti-ATR antibody. *Stands for *p* value < 0.01.

To identify the phosphatase that dephosphorylates cytoplasmic ATR at Ser428 human A549 cells were transfected with siRNAs of several phosphatases. The siRNA-knocked down cells then were UV-treated. After a brief recovery, cellular fractionation was performed and cell lysates were analyzed by WB. As shown in Figure 1A, the level pATR(Ser428) was greatly reduced in mock-treated (control) cells after UV irradiation (lanes 1 vs. 5). In the siRNA-treated cells only those with PP2Ac knockdown abolished the UV-induced dramatic reduction of pATR(Ser428) level in the cytoplasm as compared to the UV-treated control cells (lanes 5 vs. 6). Interestingly, even in the absence of UV, siRNA knockdown of PP4c and PP5 reduced pATR(Ser428) level dramatically. However, there remained a significant decrease of pATR(Ser428) after UV irradiation with siPP4c and siPP5 knockdown (lanes 5 vs. 7 and 8). Thus, siRNAs targeting of these phosphatases, had little effect on the levels of pATR(S428) in the cytoplasm. Also, the cellular fractionation demonstrates that the effect of PP2A siRNA on pATR(S428) is limited to the cytoplasm of the cell (Figure 1B).

To demonstrate the specificity of PP2A in dephosphorylating ATR at Ser 428, an ATR expression construct in which the Ser 428 residue had been mutated to alanine (S428A) was introduced into an ATR^flox/–^ cell line. As shown in Figure 1C, the mutation abolished the effect of UV-induced PP2A dephosphorylation on ATR S428 in the cytoplasm. In addition, the Duolink *in situ* proximity ligation assay (PLA) showed that PP2A interacted directly with ATR (Figure 1D) to perform its dephosphorylating activity. The increased interaction was highest at 1 h but was still significant at 2 h following UV irradiation. These results were confirmed with co-immunoprecipitation of PP2A with anti-ATR antibody (Figure 1E), indicating that PP2A did interact with the ATR to dephosphorylate phospho-S428.

### PP2A Dephosphorylates Cytoplasmic ATR at S428 Independent of ATRIP and PP2A’s Activity Is UV Dose-Dependent

**FIGURE 2:**
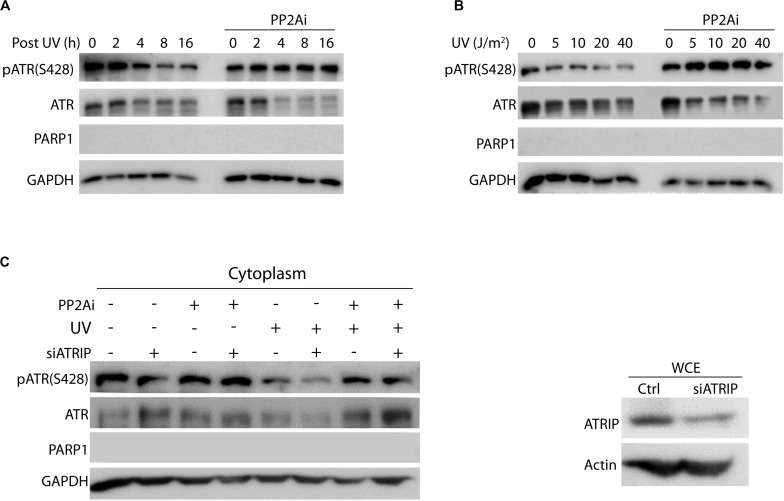
Protein phosphatase 2A (PP2A) dephosphorylates cytoplasmic pATR (S428) in a UV dose- and recovery time-dependent manner and is independent of ATRIP. **(A)** Cytoplasmic pATR (S428) dephosphorylation by PP2A is dependent on post-irradiation recovery time in A549 cells. The WB shows that the level of ATR phosphorylation at S428 depends on the recovery time following UV irradiation at 40 J/m^2^. PP2A inhibitor treatment (PP2Ai) abolishes this dephosphorylation at all recovery times. **(B)** Cytoplasmic pATR (S428) dephosphorylation by PP2A is UV dose dependent and is attenuated with PP2A inhibitor treatment of A549 cells. Control A549 cells show a reduction in the level of pATR (S428) that is dependent on the dose of UV irradiation used, but retain their level of pATR (S428). **(C)** A549 cells, depleted of ATRIP by siRNA knockdown, were divided into two groups with one treated with PP2A inhibitor and the other left as a control. WB assay shows that this ATRIP knockdown does not affect PP2A’s dephosphorylation of pATR (S428) in the cytoplasm of A549 cells. The right panel shows the efficiency of the ATRIP knockdown.

It is well known that ATR’s nuclear function in the DNA damage signaling is dependent on ATRIP. To determine if PP2A dephosphorylation of cytoplasmic pATR(S428) and, therefore, the formation of *cis* ATR requires ATRIP, ATRIP-knockdown cells were UV-treated, then fractionated and the cytoplasmic fraction analyzed by WB (Figure 2). The level of pATR(S428) was increased with PP2A inhibitor treatment in both the presence and absence of ATRIP (Figure 2C). Also, we observed no significant difference in dephosphorylation of pATR(S428) by PP2A in the cytoplasm between cells with or without ATRIP depletion (Figure 2C). This suggests that in the absence of ATRIP, the nuclear binding partner of ATR, PP2A in the cytoplasm was still able to dephosphorylate ATR at S428. This is consistent with ATRIP depletion having no effect on cytoplasmic ATR-H formation and its anti-apoptotic function at the mitochondria (Hilton et al., 2016). In addition, to investigate whether the activity of PP2A was dependent on post-irradiation recovery time or UV dose, cells were UV treated and allowed to recover for 0–16 h or irradiated at different doses of UV followed by a 2 h recovery before cellular fractionation and WB analysis. The UV-induced depletion of cytoplasmic pATR (S428) (shown in Figure 1B) occurred in both a recovery time- (Figure 2A) and a dose- (Figure 2B) dependent manner. This is consistent with the earlier reported data that ATR-H (*cis* ATR) formation in the cytoplasm was UV dose- and post-UV recovery time-dependent given that Ser428 dephosphorylation promotes ATR-H formation (Hilton et al., 2016). However, PP2A inhibitor reversed the pattern by significantly increasing the pATR level in both time- and dose-dependent manners, likely leading to the relatively more ATR-L formation than ATR-H (Hilton et al., 2016).

### PP2A Dephosphorylation of ATR-L (*trans-*ATR) Promotes the Formation of ATR-H (*cis* ATR) in the Cytoplasm

**FIGURE 3:**
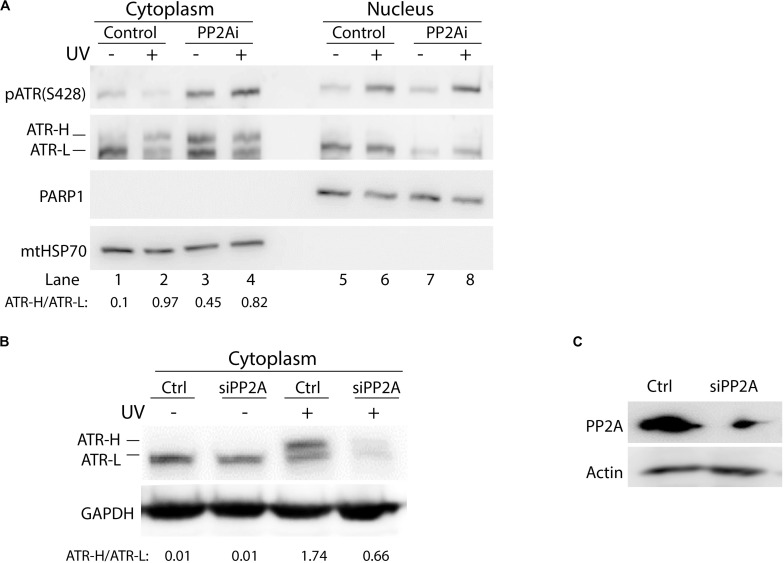
Protein phosphatase 2A (PP2A) dephosphorylation of pATR changes ATR to the higher form (ATR-H). **(A)** UV irradiation of A549 cells at 40 J/m^2^ increases the formation of ATR-H in the cytoplasm. Also, the ATR-H is not phosphorylated while the ATR-L band is phosphorylated in both the nucleus and the cytoplasm (cytoplasm to nucleus loading ratio = 3:1). The samples were analyzed on a 3–8% gradient gel. **(B)** A549 cells were pretreated with DMSO (Ctrl) or transfected with PP2A siRNAs, then UV-irradiated at 40 J/m^2^ (–/+UV) followed by a 2 h recovery. The cytoplasmic fraction was analyzed by electrophoresis through a 3–8% gradient gel and WB to show the form of ATR in the cytoplasm. PP2A depletion reduces the levels of cytoplasmic ATR and the amount of ATR-H formed following UV treatment. **(C)** WB showing PP2A siRNA knockdown efficiency in A549 cells.

To investigate whether phosphorylation of cytoplasmic ATR at S428 affects the formation of ATR-H (*cis* ATR) and ATR-L (*trans* ATR) in the cytoplasm, PP2A was knocked down by PP2A siRNA, followed by UV irradiation of the cells, cellular fractionation, 3–8% gradient gel electrophoresis and WB. In the control cells, as expected, pATR (S428) levels were reduced with UV damage as compared to that without UV (Figure 3A). More ATR was found in ATR-L form relative to the ATR-H form in the cells in the absence of UV treatment, while the level of ATR-H relative to ATR-L increased and accumulated in UV-treated cells (Figures 3A,B). The latter is due to the UV-induced inhibition of Pin1 activity (Hilton et al., 2016). However, ATR-H accumulation is reversed by PP2A depletion, which kept ATR in the *trans* form in non-irradiated cells, and also promoted *trans* ATR formation in UV-irradiated cells (Figures 3B,C). It should be noted that in Figure 3A, PP2A inhibition resulted in an unexpected amount of ATR-H in the cytoplasm of the cells without UV treatment. This is probably due to the known non-specific activity of LB-100 relative to the siRNA treatment, which is much more specific and showed no such effects (Figure 3B). Despite this, the patterns for the data of Figures 3A,B remain the same as either PP2A inhibition or siRNA depletion decreases the ATR-H/ATR-L ratios in UV-treated cells, while UV increases the ratio in cells without PP2A inhibition or depletion.

It is worth noting that in cells without PP2Ai treatment, UV irradiation consistently reduced cytoplasmic pATR (Ser428) (Figure 1B), but increased pATR (Ser428) in the nucleus. These observations suggest that phosphorylation of ATR on Ser428 is regulated differently in the cytoplasm and the nucleus of cells in response to DNA damage. This further confirms that ATR functions in the nucleus differ significantly from its roles in the cytoplasm.

### Inhibition of PP2A Reduces the Association of ATR-H With tBid at Mitochondria

**FIGURE 4:**
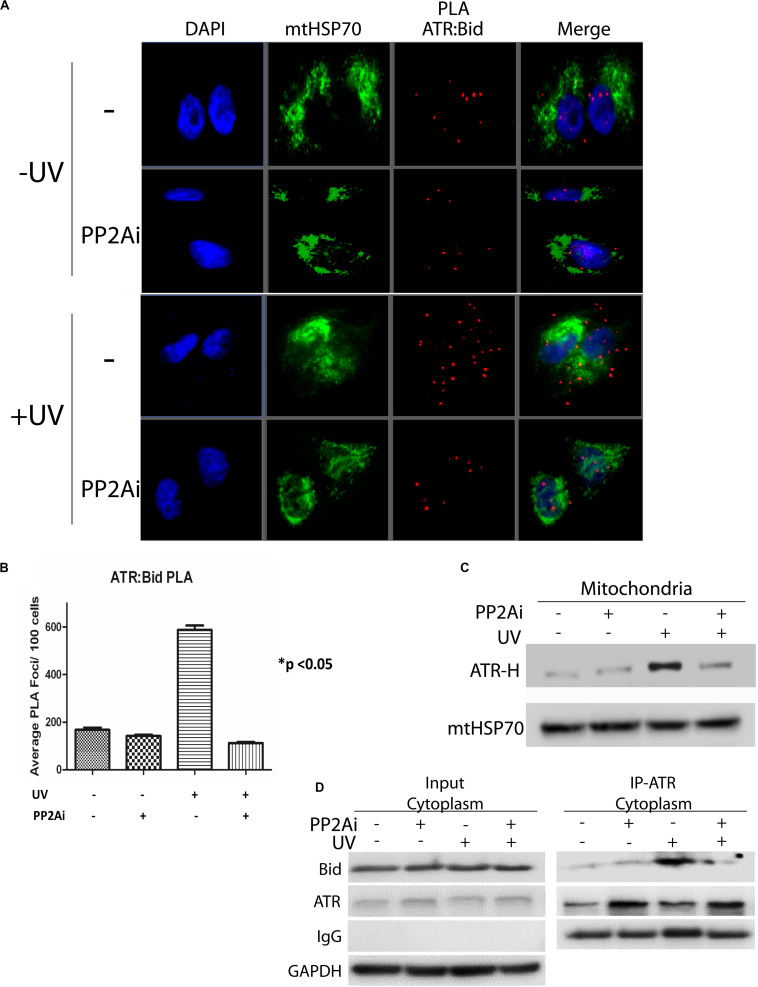
Inhibition of PP2A’s dephosphorylation of pATR and ATR-L accumulation in the cytoplasm leads to reduced association of ATR with tBid on mitochondria. **(A)** PLA shows that cytoplasmic ATR directly associates with Bid and that this association increases with UV treatment, but the UV-induced association is attenuated by PP2A inhibitor treatment. The nuclei are stained with DAPI and mitochondria are indicated by mtHSP70 immunofluorescence. A549 cells were UV irradiated at 40 J/m^2^ with a 2 h recovery. **(B)** A graphic display of the PLA data shown in **(A)**. The *p* < 0.05 refers to the sample (+UV, −PP2Ai) versus any of other samples. **(C)** ATR-H accumulation at the mitochondria increases with UV treatment at 40 J/m^2^ in A549 cells, but is reduced in cells treated with PP2A inhibitor. **(D)** Biochemical confirmation of the reduction in the cytoplasmic ATR:tBid association observed by PLA **(A,B)**. A549 cells were pretreated with PP2Ai followed by UV irradiation with a 2 h recovery. After cellular fractionation ATR was immunoprecipitated from the cytoplasmic fraction using C-terminal-specific ATR antibody. The association of tBid with ATR was confirmed by WB.

We reported previously that upon UV irradiation ATR-H directly interacts with proapoptotic protein tBid at mitochondria, blocking Bax-tBid interaction and thus preventing activation of apoptosis (Hilton et al., 2016). Given that PP2A dephosphorylates pATR-L (*trans*-ATR) at Ser428 and, thus, regulates ATR-H formation, we determined the effects of PP2A inhibition on this ATR-tBid interaction. We examined the ATR-tBid binding in two ways. First, using the Duolink *in situ* proximity ligation assay (PLA) we demonstrated that the UV-induced direct interaction between ATR and tBid is significantly reduced when PP2A is inhibited (Figures 4A,B). This finding was corroborated by isolating mitochondria from UV-irradiated cells with or without PP2A inhibition (Figure 4C). The results show that there is significantly less ATR-H at mitochondria when the PP2Ai is present. The inhibition significantly reduced the amount of mitochondria-associated ATR-H (Figure 4C). We further confirmed these findings with a co-immunoprecipitation (co-IP) assay of tBID-ATR complexes in the cytoplasmic fraction of treated cells (Figure 4D). The immunoprecipitated ATR from PP2A-inhibited UV-irradiated cells had less associated tBid protein than that from non-inhibited cells. These data suggest that PP2A inhibition would result in increased cell death since ATR-H needs to be associated with tBID at mitochondria for it to exert its anti-apoptotic function (Hilton et al., 2016).

### Inhibition of PP2A Decreases Cellular Viability in Response to UV-Induced DNA Damage

**FIGURE 5:**
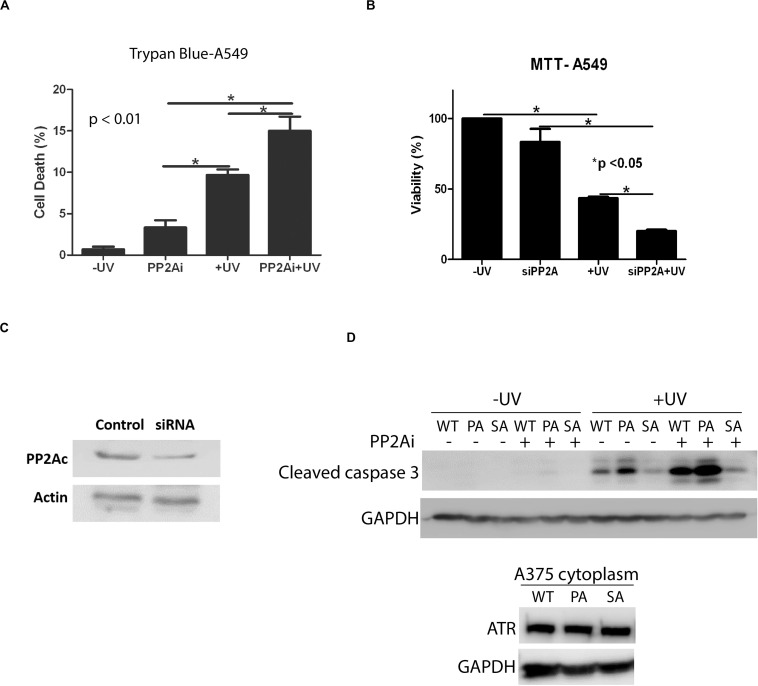
Inhibition of PP2A decreases cellular viability which is enhanced UV-induced DNA Damage. **(A)** A trypan blue exclusion assay shows that PP2A depletion with siRNA knockdown increases apoptosis in A549 cells and that the level of apoptosis is increased further by UV irradiation at 40 J/m^2^ with a 24 h recovery time. **(B)** MTT assay showing increased levels of apoptosis in A549 cells depleted of PP2A by siRNA knockdown which is enhanced by UV irradiation at 40 J/m^2^ and a 24 h recovery time. **(C)** WB showing PP2A siRNA knockdown efficiency in A549 cells. **(D)** WB shows the caspase 3 cleavage assay which indicates the apoptosis activation, in transgenic A375 human melanoma cells expressing wild type ATR (WT), ATR-P429A (PA) or ATR-S428A (SA) 24 h following UV irradiation in the presence and absence of PP2Ai treatments. *Stands for *p* value < 0.01 or 0.05.

Next, we investigated the effect of PP2A inhibition on the apoptotic cell death induced by UV. Figure 5A shows that depletion of active PP2A led to a significant increase in A549 cell death at 24 h following UV damage in comparison with the cells without PP2A inhibition. The results of trypan blue exclusion assay also were confirmed using the colorimetric MTT assay (Figure 5B). Note that PP2A depletion, either by inhibition or by knockdown, had a smaller but significant effect on cell viability without UV irradiation. Furthermore, we assess the cytoplasmic cleaved caspase-3 levels in transgenic knock-in ATR-S428A and ATR-P429A human melanoma cells (A375 cells) (Figure 5D). Opposite effects on apoptosis are observed between ATR-S428A and ATR-P429A cells because the ATR-P429A cells contained only *trans* ATR, and thus, there was little or no *cis* ATR (*cis* ATR null) in the cells. Since *cis* ATR is antiapoptotic, this means ATR-P429A is pro-apoptotic and ATR-P429A cells are more sensitive to apoptotic signals than wild-type A375 cells, thus resulting in greater cell death. In contrast, ATR-S428A cells contain an overwhelming amount of antiapoptotic *cis* ATR in the cytoplasm which significantly reduced the apoptotic activity. Together, these results strongly suggest that the effects of PP2A dephosphorylation of ATR at Ser428 may have a direct impact on the DNA damage-induced cell death.

## Discussion

**FIGURE 6:**
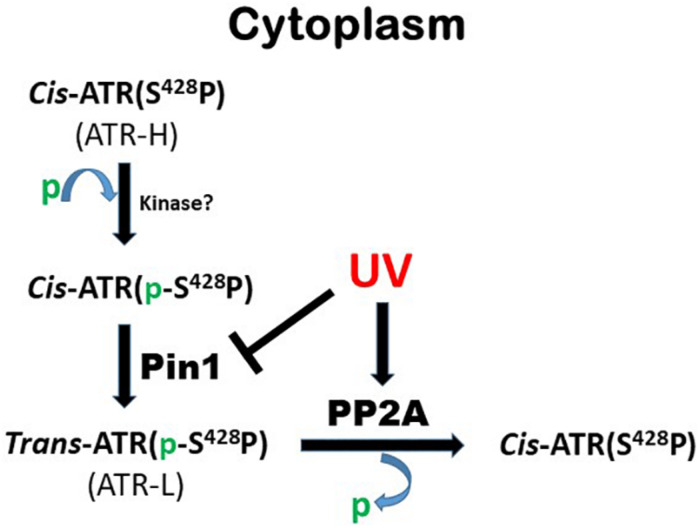
Summary of role of PP2A in Pin1-mediated ATR isomerization in the cytoplasm.

As summarized in Figure 6, this work identifies PP2A as the phosphatase that dephosphorylates ATR at Ser428 following UV-induced DNA damage. PP2A mediated the reduction of pATR (S428) in the cytoplasm following UV treatment (Figure 1A). Since Pin1’s activity requires the Ser428-Pro429 recognition site in ATR to be phosphorylated, PP2A antagonizes Pin1’s action by depleting the amounts of cytoplasmic pATR at S428. This PP2A control over the phosphorylation status of ATR at the Pin1 recognition motif adds another level of regulatory complexity to the DDR process. Following DNA damage, PP2A interacts with ATR (Figures 1D,E) to dephosphorylate its Pin 1 recognition motif to prevent the further isomerization of *cis* to *trans* ATR in the cytoplasm, resulting in *cis* ATR-H accumulation. In addition, PP2A’s dephosphorylation of ATR at S428 is ATRIP independent. This further highlights how unique and distinct cytoplasmic ATR is from its nuclear counterpart which requires its partner ATRIP to perform its kinase activities following DNA damage. We found that PP2A activity led to the accumulation of *cis* ATR-H in the cytoplasm and, then, at the mitochondria following DNA damage (Figure 4C). Mitochondrial *cis* ATR-H is an antiapoptotic protein that binds to tBid (Figures 4A,B,D) to prevent activation of apoptosis and to suppress DNA damage-induced apoptotic cell death (Figures 5A–C). It should be noted that PP2A inhibition appears to reduce the ATR protein level in cells treated with UV (Figure 2A,B). It is possible that the decrease in ATR level could be due to the degradation of ATR in the cells undergoing apoptosis caused by depleted PP2A activity. Importantly, however, the pATR level that signals the Pin1-mediated ATR isomerization pathway was significantly increased, leading to relatively more ATR-L formation than ATR-H (Hilton et al., 2016).

Protein phosphatase 2A depletion led to reduced levels of UV-induced antiapoptotic ATR-H in the cytoplasm. While this is true, the study also showed that a marginal level of ATR-H remained in the cytoplasm or at mitochondria, even after PP2A depletion (Figure 3B). This is consistent with our previous observation that UV can inactivate Pin1 via DAPK1 phosphorylation of Pin1 at Ser71 (Hilton et al., 2016). Therefore, a combination of effects from Pin1-Ser71 phosphorylation by DAPK1 (Lee et al., 2011) and PP2A dephosphorylation of ATR at Ser428 may result in the ultimate formation of antiapoptotic *cis* ATR-H at mitochondria. Unfortunately, the kinase(s) for phosphorylation of ATR-Ser428 remains unidentified. Overall, these processes should be considered as possible candidates in developing new targets for adjuvant chemotherapy.

Apoptosis is an important cellular process that is tightly regulated by the cell, and in the cytoplasm a balance between ATR-H and ATR-L levels needs to be maintained for proper homeostasis. Pin1 is central to the regulation of this balance since it catalyzes the phosphorylation-dependent isomerization that converts *cis* ATR-H to *trans* ATR-L in the cytoplasm. The proper balance ensures a functional and effective DNA damage response as the protection of cells from apoptosis is essential for the activities of cell cycle checkpoint arrest and DNA repair. Our findings reported in this study suggest that PP2A plays an important role in regulating this balance.

Our results reveal a new mechanism by which the cell can regulate mitochondrial apoptosis. Because deregulated apoptosis is a key hallmark in carcinogenesis, the involvement of PP2A in the regulation suggests the importance of the phosphorylation status of cytoplasmic Ser428 of ATR in carcinogenesis and cancer treatment. Indeed, this is supported by the significant correlation of the dephosphorylation status of cytoplasmic ATR (Ser428) with the severity of ovarian cancer in patients such as advanced stage, serous histology, large residual mass and so on (Lee et al., 2015).

Our findings indicate that ATR-mediated regulation of the mitochondrial cell death pathway can be altered by changing the phosphorylation status of pATR at S428. Because the PP2A regulation of pATR (S428) status is localized to the cytoplasm, modulating this ATR phosphorylation should have minimal effects on the DNA damage-signaling pathway in the nucleus. This defines PP2A as a potential target in regulating ATR’s anti-apoptotic activity. It is well documented that PP2A is a heterotrimeric protein of various subunit isoforms, particularly the regulatory subunit B, each with distinct functions (Janssens and Goris, 2001; Cho and Xu, 2007; Seshacharyulu et al., 2013; Sangodkar et al., 2016; Wlodarchak and Xing, 2016; Reynhout and Janssens, 2019). To achieve a specific inhibition of PP2A for its activity at ATR (Ser428) to reduce potential adverse side effects, identification of the regulatory subunit isoform specifically responsible for ATR (Ser428) dephosphorylation is necessary though it is out of scope of this study.

In summary, our results have allowed a better understanding of the mechanism of how ATR’s anti- apoptotic activity at the mitochondria is regulated. This also opens up the possibilities of targeting PP2A for potential translational applications.

## Data Availability Statement

All datasets generated for this study are included in the article/supplementary material.

## Author Contributions

YM performed most of the experiments and wrote the draft of the manuscript. BC started the project and generated the data during the early period of the study. HB performed some of the experiments during manuscript revision. PM participated in experimental design, data analysis, and manuscript preparation. ZL provided help to the experiments. YZ is the senior author who oversaw and directed this study from the beginning through the manuscript preparation. All authors contributed to the article and approved the submitted version.

## Conflict of Interest

The authors declare that the research was conducted in the absence of any commercial or financial relationships that could be construed as a potential conflict of interest.
